# YY1 mediates TGF-β1-induced EMT and pro-fibrogenesis in alveolar epithelial cells

**DOI:** 10.1186/s12931-019-1223-7

**Published:** 2019-11-08

**Authors:** Chuyi Zhang, Xiaoping Zhu, Yifei Hua, Qian Zhao, Kaijing Wang, Lixiao Zhen, Guangxue Wang, Jinhui Lü, An Luo, William C. Cho, Xin Lin, Zuoren Yu

**Affiliations:** 1 Research Center for Translational Medicine, Shanghai East Hospital, Tongji University School of Medicine, 150 Jimo Road, Shanghai, 200120 China; 2 Shanghai Institute of Heart Failure, Shanghai East Hospital, Tongji University School of Medicine, 150 Jimo Road, Shanghai, 200120 China; 3 Department of Respiratory Medicine, Shanghai East Hospital, Tongji University School of Medicine, 150 Jimo Road, Shanghai, 200120 China; 4 Department of Clinical Oncology, Queen Elizabeth Hospital, Kowloon, Hong Kong, China

**Keywords:** YY1, Pulmonary fibrosis, EMT, alveolar epithelial cells

## Abstract

Pulmonary fibrosis is a chronic, progressive lung disease associated with lung damage and scarring. The pathological mechanism causing pulmonary fibrosis remains unknown. Emerging evidence suggests prominent roles of epithelial–mesenchymal transition (EMT) of alveolar epithelial cells (AECs) in myofibroblast formation and progressive pulmonary fibrosis. Our previous work has demonstrated the regulation of YY1 in idiopathic pulmonary fibrosis and pathogenesis of fibroid lung. However, the specific function of YY1 in AECs during the pathogenesis of pulmonary fibrosis is yet to be determined. Herein, we found the higher level of YY1 in primary fibroblasts than that in primary epithelial cells from the lung of mouse. A549 and BEAS-2B cells, serving as models for type II alveolar pulmonary epithelium in vitro, were used to determine the function of YY1 during EMT of AECs. TGF-β-induced activation of the pro-fibrotic program was applied to determine the role YY1 may play in pro-fibrogenesis of type II alveolar epithelial cells. Upregulation of YY1 was associated with EMT and pro-fibrotic phenotype induced by TGF-β treatment. Targeted knockdown of YY1 abrogated the EMT induction by TGF-β treatment. Enforced expression of YY1 can partly mimic the TGF-β-induced pro-fibrotic change in either A549 cell line or primary alveolar epithelial cells, indicating the induction of YY1 expression may mediate the TGF-β-induced EMT and pro-fibrosis. In addition, the translocation of NF-κB p65 from the cytoplasm to the nucleus was demonstrated in A549 cells after TGF-β treatment and/or YY1 overexpression, suggesting that NF-κB-YY1 signaling pathway regulates pulmonary fibrotic progression in lung epithelial cells. These findings will shed light on the better understanding of mechanisms regulating pro-fibrogenesis in AECs and pathogenesis of lung fibrosis.

Pulmonary fibrosis occurs primarily in older adults, and limited to the lungs. It is characterized by progressive worsening of dyspnea and interstitial infiltrates in lung parenchyma. The progressive fibrosis is always associated with epithelial to mesenchymal transition (EMT) of alveolar epithelial cells (AECs), failed regeneration of normal alveolar structure, and activated fibroblasts [1]. Revealing the mechanisms by which AECs maintain homeostasis or contribute to fibrosis may be of help in discovering novel targets to prevent and/or treat pulmonary fibrosis.

Although it is believed that lung fibrosis is related with multiple factors including viral infection, smoking and/or environmental exposures to pollutants, toxic dusts, etc., the pathological mechanisms remain unclear. Emerging evidence demonstrated the contribution of damaged lung epithelium to fibrosis, such as epithelial micro injuries and abnormal wound healing [2, 3]. Moreover, myofibroblasts are believed to play a crucial role in the pathogenesis of pulmonary fibrosis [4]. Endogenous lung fibroblasts, circulating bone marrow-derived fibrocytes and EMT of AECs have been demonstrated as the main origin of myofibroblast [5–7]. Myofibroblasts express contractile proteins, such as α-smooth muscle actin (α-sma), and produce large amounts of matrix proteins [8]. Macrophage and lymphocyte subpopulations also regulate pulmonary fibrosis by releasing fibrogenic growth factors [9].

Serving as a kind of “multipotent” progenitor cells with considerable plasticity, AECs have potential to regenerate normal alveolar architecture through re-epithelialization or transdifferentiate to fibroblasts through EMT [10, 11]. There are two types of AECs in the lung, type I and type II. type II AECs constitute ~ 60% of alveolar epithelial cells and 10–15% of all lung cells, covering ~ 5% of the alveolar surface area [12]. Bleomycin accelerates the transdifferentiation of Type II into Type I AECs, which can be impaired by hyperoxia [13]. Keratinocyte growth factor is capable of partially reversing transdifferentiation between Type II and Type I phenotypes in primary culture, suggesting plasticity between the two types of AECs [14]. Type II AEC-derived cell lines are frequently reported to undergoing EMT. The excessive proliferation and hypertrophy of Type II AECs have been demonstrated to involve in regulation of pulmonary fibrosis [15, 16]. R3/1 is a cell line belonging to alveolar type I epithelial cells. As adenocarcinomic alveolar basal epithelial cells, A549 has served as a model of alveolar Type II-like pulmonary epithelium [17]. As a bronchial epithelial cell line, BEAS-2B also undergoes EMT and contributes to the pulmonary fibrosis pathology in a given situation [1].

Transforming Growth Factor (TGF)-β has been considered as a key molecule in activation of the fibrotic program. TGF-β is upregulated and activated in fibrotic diseases. It modulates fibroblast phenotype and function through inducing myofibroblast transdifferentiation, inducing EMT of AECs and promoting matrix preservation [8, 18]. Overexpression of TGF-β led to pulmonary fibrosis in mice with minimal inflammation [19]. TGF-β induces renal fibrosis via activation of both canonical and non-canonical signaling pathways, resulting in activation of myofibroblasts, overexpression of extracellular matrix and inhibition of ECM degradation [20].

Yin Yang1 (YY1), as an important transcription factor, is a member of the GLI-Krüppel class proteins [21]. YY1 plays crucial role in numerous biological processes by selectively activating or repressing gene transcription, depending upon promoter contextual differences and specific protein interactions. Aberrant expression of YY1 is frequently observed in a large number of cancers, such as colon, gastric and breast cancers [22]. The overexpression of YY1 in the majority of cancers has been correlated with poor prognosis.

EMT in cancer entails the molecular reprogramming and phenotypic changes that characterize the conversion of immobile cancer epithelial cells to motile mesenchymal cells. YY1 has been demonstrated to induce EMT of cancer cells, having potential as a novel prognostic biomarker for EMT and/or therapeutic target for prevention of metastasis [23]. In addition to regulating EMT, YY1 overexpression is correlated with tumor progression and drug resistance in prostate cancer [24]. YY1 also regulates fibrotic lung diseases [25]. YY1 is overexpressed in fibroblasts in both human pulmonary fibrosis and murine fibrotic models, regulating fibrogenesis at least in part by increasing α-sma and collagen expression [25]. However, the specific function of YY1 in lung epithelial cells during the pathogenesis of lung fibrosis is yet to be determined.

Herein, EMT and pro-fibrotic phenotypes were induced in A549 and BEAS-2B cells by TGF-β treatment, in which YY1 was demonstrated to mediate the TGF-β-induced EMT phonotype. The cytoplasm to the nucleus translocation of NF-κB p65 was associated with TGF-β-activated fibrosis signaling. These findings will shed light on the better understanding of mechanisms through which EMT in AECs leads to pathogenesis of pulmonary fibrosis.

## Materials and methods

### Cell lines and culture

R3/1, A549 and BEAS-2B cells were purchased from ATCC and maintained in our lab. The culturing condition includes DMEM medium containing 10% fetal bovine serum (FBS) and penicillin (100 U/ml) / streptomycin (100 μg/ml). To induce EMT and fibrosis, serum-free DMEM medium containing 5 or 20 ng/ml of TGF-β (Gibco, PHG9214) was used.

### Animals

8-week-old C57BL/6 J and BALB/C mice were purchased from Silaike animal company (Shanghai, China). Animal studies were approved by the Institutional Animal Care and Use Committee of the Tongji University School of Medicine. Isolation of the epithelial and fibroblast cells from the lung of mouse were performed in the Research Center for Translational Center, Tongji University School of Medicine. All experiments were performed in accordance with the relevant guidelines and regulations for animal use.

### Lentivirus production and infection

Lentiviral production and infection methods have been described previously in detail [25]. Briefly, the lentiviral particles were produced by transient co-transfection of HEK293T cells with the packaging plasmids psPAX2 and pMD2.G and pLKO.1 -YY1 shRNA. Virus were harvested in 48 h after transfection. Cells were infected with the virus adding into fresh media containing polybrene (8 μg/ml). The infected cells were cultured for use after screening with puromycin.

### Plasmid transfection

YY1 mRNA coding sequence was amplified and inserted into pSG5 vector to generate YY1-overexpressing plasmid. Empty vector was used as control. Lipofectamine 2000 (Invitrogen, USA) was used to perform transfection following the manufacturer’s instructions.

### QRT-PCR analysis

Total RNA was extracted with Trizol reagent (Invitrogen). The ABI 7900 HT Sequence Detection System (Applied Biosystem, Life Technologies, USA) was used for QRT-PCR analysis. Primers were designed using on-line Primer Design Tool from GenScript. The primer sequences were available upon request. GAPDH was used for normalization.

### Immunofluorescence staining

Adherent cells were fixed with 4% paraformaldehyde for 15 min, permeabilized with 0.1% Triton X-100 and then blocked with 1% BSA for 1 h. After incubation with primary antibody (1:200) overnight at 4 °C, FITC-conjugated goat anti rabbit IgG (ab6717, Abcam, 1:200) was used as secondary antibody. 6-diamidino-2-phenylindole (DAPI) was used for nuclear counterstaining. The slides were photographed using fluorescence microscopy (Leica, Germany). Primary antibodies were: anti-α-sma (ab5694, Abcam), anti-NF-κB p65 (D14E12, cell signaling).

### Western blot

Cell lysates (50 μg) were separated by 10% SDS-PAGE. The proteins were transferred to PVDF membrane. After blocking in 5% milk (w/v) at room temperature for 1 h, the membranes were incubated at 4 °C overnight with primary antibodies (1:1000). Following PBST washing for three times with 5 min for each, the membranes were incubated with secondary antibodies (1:7500) at room temperature for 1 h followed by washing and staining. The antibodies were: anti-β-actin (sc-47,778, Santa Cruz Biotechnology), anti-YY1 (sc-1703, Santa Cruz Biotechnology), anti- NF-κB p65 (D14E12, cell signaling), anti-histone (AH433–1, Beyotime, China), anti-E-cadherin (sc-7870, Santa Cruz Biotechnology), anti-vimentin (sc-32,322, Santa Cruz Biotechnology), anti-snail1 (sc-271,977, Santa Cruz Biotechnology) and anti-GAPDH (CST 5174, Cell Signaling Technology).

### Isolation of epithelial and fibroblast cells from mouse lung

The method for isolation of AECs was established by adaption and modification of several previous publication [26]. Briefly, after anesthetization of a mouse, the distal lung was collected and cleared with 1 x PBS after removing trachea and bronchia. The lung was cut into small pieces, digested using trypsin (1 mg/ml in PBS) containing 0.01% deoxyribonuclease I for 5 min at 37 °C, followed by digestion with collagenase type I (Sigma c0130) (1 mg/ml in PBS) containing 0.01% deoxyribonuclease I for 15 min at 37 °C. The cell suspension was filtered through a 40 μm cell strainer (BD Biosciences) to obtain the single cell suspension, and cultured in DMEM medium. Differential adhesion method was applied to purify the pulmonary epithelial cells. For isolation of fibroblasts, the lung pieces (around 1 mm × 1 mm × 1 mm in size) were seeded in T25 cell culturing flask. After culturing for 3 h with upside down, turning the flask back to its right position to continue culturing until a single layer of cells were grown. The fibroblasts were purified through differential adhesion method. The attached cell layer was trypsinized to single cell suspension, then plated onto a tissue culture flask for incubation for 40 min. The cells adhered to the substratum are largely fibroblastic in nature.

### Statistical analysis

Data are presented as mean ± SEM. The standard two-tailed student’s *t*-test was used for statistical analysis, in which *p* < 0.05 was considered statistical significance.

## Results

### Upregulation of YY1 by TGF-β treatment in A549 cells

**Fig. 1:**
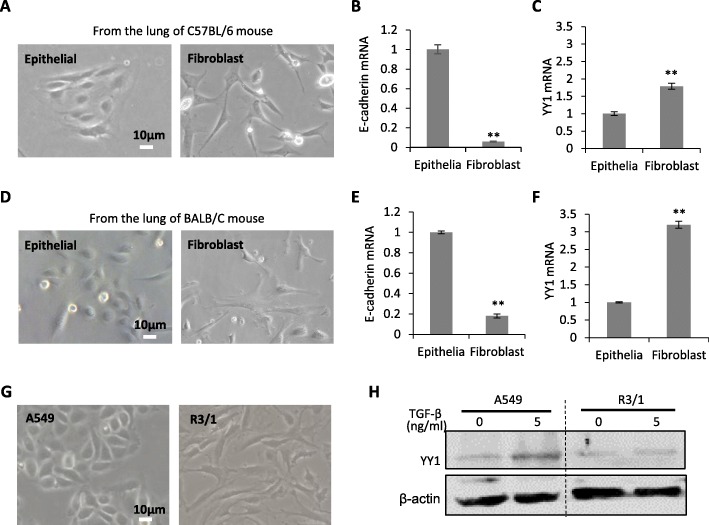
YY1 expression in alveolar epithelial cells. **a** The fibroblast cells and alveolar epithelial cells isolated from the fresh lung of C57BL/6 mouse. Scale bar: 10 μm. **b** and **c** Quantitative RT-PCR analysis of E-cadherin (**b**) and YY1 (**c**) expression in the fibroblast and epithelial cells in **a**. **d** The fibroblast cells and alveolar epithelial cells isolated from the fresh lung of BALB/C mouse. Scale bar: 10 μm. **e** and **f** Quantitative RT-PCR analysis of E-cadherin (**e**) and YY1 (**f**) expression in the fibroblast and epithelial cells in **d**. **g** Morphology of A549 and R3/1 lung epithelial cells. Scale bar: 10 μm. **h** Western blot analysis of YY1 in A549 and R3/1 cells with or without TGF-β treatment for 24 h. GAPDH served as housekeeping gene for normalization in Quantitative RT-PCR assays. β-actin served as loading control for western blots. Data are presented as mean ± SEM (*n* = 3), **p < 0.01

In order to determine the mechanism regulating the pathogenesis of pulmonary fibrosis, the primary fibroblast cells and alveolar epithelial cells were isolated from the lung from either C57BL/6 mouse with Th1 immune response (Fig. 1a-c) or BALB/C mouse with Th2 immune response (Fig. 1d-f). The cells were further validated by gene expression analysis of epithelial marker E-cadherin. YY1, a transcriptional factor closely related with fibroblast differentiation, showed higher levels in the primary fibroblast cells than that in the primary alveolar epithelial cells with fold change around 3 in BALB/C mouse while ~ 1.5 in C57BL/6 mouse (Fig. 1c and f).

Further analysis on type I alveolar epithelial cells R3/1 and type II-like A549 cells indicated the fibrocyte-like morphology with long fusiform shape for R3/1 cells (Fig. 1g). 5 ng/ml of TGF-β was applied to both R3/1 and A549 cells to induce EMT. Western blot analysis demonstrated the induction of YY1 in expression by TGF-β treatment for 24 h in A549 cells, but not in R3/1 cells (Fig. 1h).

### Upregulation of YY1 was associated with TGF-β induced EMT and pro-fibrotic phenotypes in human lung epithelial cells

**Fig. 2:**
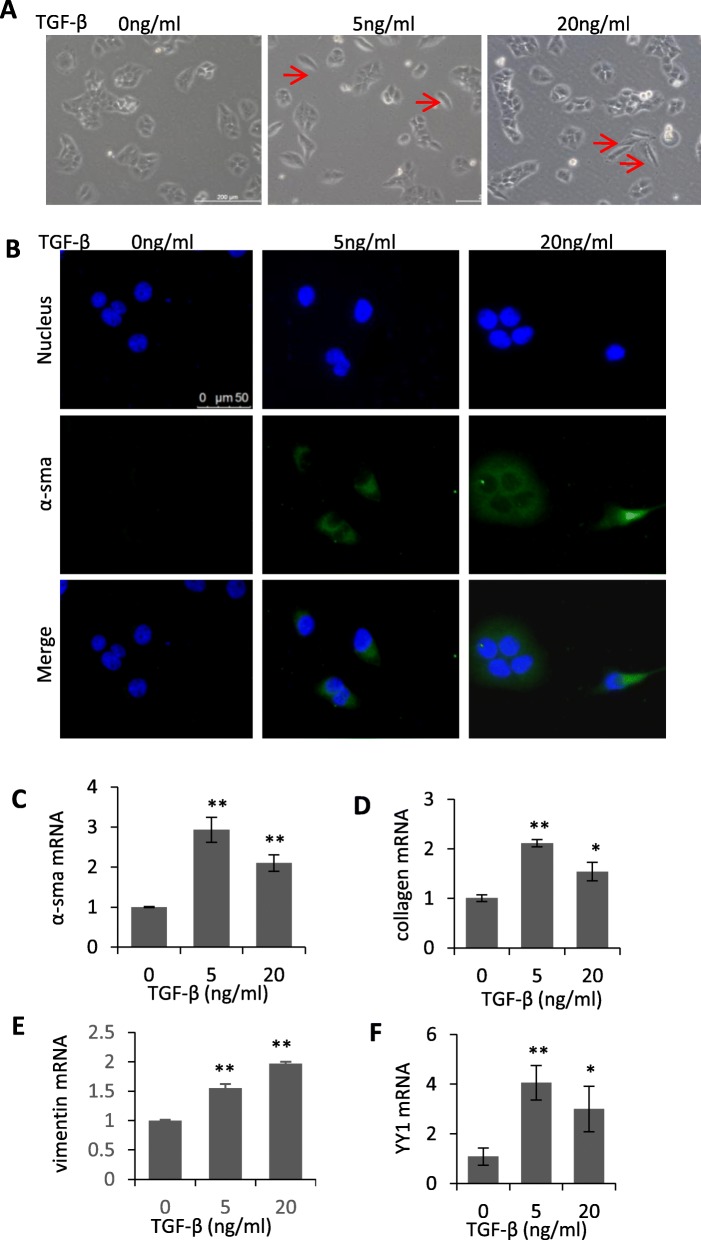
Upregulation of YY1 was associated with the TGF-β induced EMT and pro-fibrotic changes in A549 cells. **a** Morphological change of A549 cells induced by different concentrations of TGF-β. The arrows indicated representative profibrotic cells. Scale bar: 200 μm. **b** Immunofluorescence staining demonstrated the induction of α-sma by 0, 5 and 20 ng/ml of TGF-β treatment. Scale bar: 50 μm. **c**-**f** Quantitative RT-PCR analysis of mesenchymal and pro-fibrotic markers including α-sma (**c**), collagen (**d**), vimentin (**e**) and YY1 (**f)** in A549 cells before or after treatment with 5 and 20 ng/ml of TGF-β. TGF-β was applied for 48 h. Data are presented as mean ± SEM (*n* = 3). All comparisons were made with TGF-β-treated cells vs untreated cells. *p < 0.05, **p < 0.01

Different concentrations of TGF-β were used to treat A549 cells to induced pro-fibrogenesis and EMT. As shown in Fig. 2a, either 5 ng/ml or 20 ng/ml of TGF-β treatment induced the EMT-like morphological change of cells. Immunofluorescence staining demonstrated the induction of α-sma by TGF-β treatment (Fig. 2b). The TGF-β induced pro-fibrotic change was further confirmed by quantitative analysis of fibrotic biomarkers α-sma (Fig. 2c) and collagen (Fig. 2d), mesenchymal biomarkers vimentin (Fig. 2e) and slug (Additional file 1: Figure S1A), and tight junction-related biomarkers claudin-1 (Additional file 1: Figure S1B) and ZO-1 (Additional file 1: Figure S1C). Upregulation of YY1 was observed following the TGF-β induced EMT (Fig. 2f). Notably, the expression levels of EMT markers did not show significant difference between 5 ng/ml and 20 ng/ml of TGF-β concentration, suggesting 5 ng/ml TGF-β is sufficient to induce EMT-like phenotype in the lung epithelial cells.

### YY1 mediated TGF-β-induced EMT and pro-fibrotic phenotypes in human lung epithelial cells

**Fig. 3:**
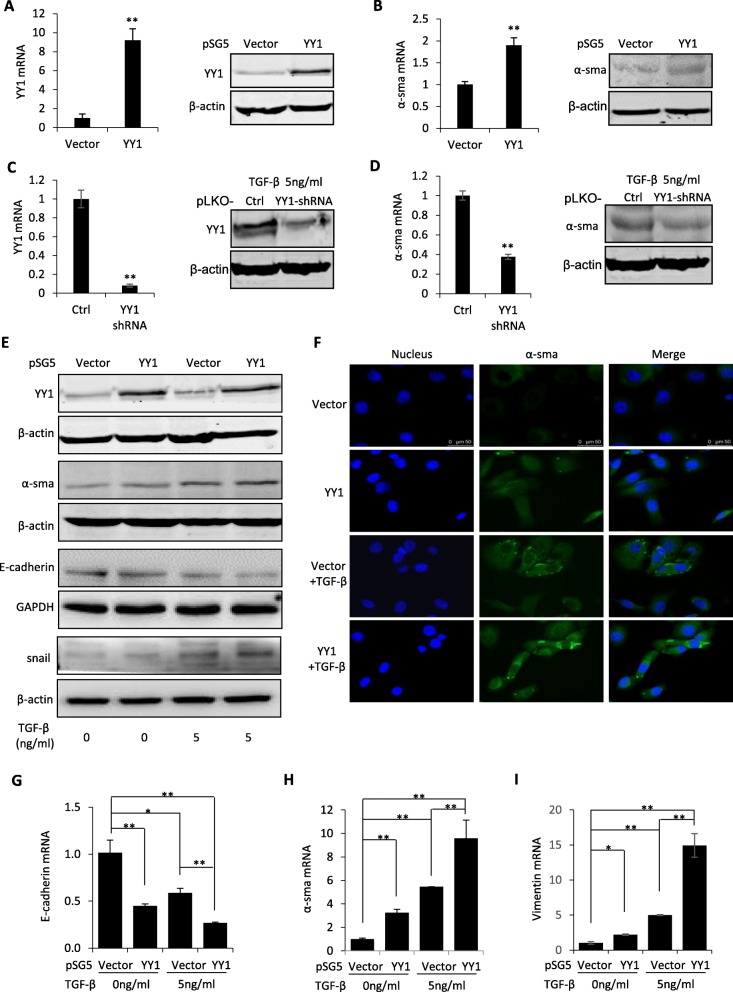
YY1 mediated TGF-β-induced EMT and pro-fibrotic phenotypes. **a**, **b** QRT-PCR and western blot were applied to analyze YY1 (**a**) and α-sma (**b**) expression at the mRNA and protein levels in A549 cells transfected with YY1-overexpressing plasmid or pSG5 vector control. **c**, **d** QRT-PCR and western blot were applied to analyze YY1 (**c**) and α-sma (**d**) expression at the mRNA and protein levels in A549 cells transfected with YY1-shRNA or pLKO vector control. **e** Proteomic analyses of α-sma, YY1, E-cadherin and snail in A549 cells treated by 0 or 5 ng/ml of TGF-β with or without overexpression of YY1. **f** Immunofluorescence staining of α-sma in A549 cells treated by 0 or 5 ng/ml of TGF-β with or without overexpression of YY1. Scale bar: 50 μm. **g**-**i** Primary alveolar epithelial cells isolated from the lung of C57BL/6 J mice were applied to perform QRT-PCR analyses on the expression of E-cadherin (**g**), α-sma (**h**) and vimentin (**i**) under treatment of 0 or 5 ng/ml of TGF-β with or without overexpression of YY1. β-actin or GAPDH served as loading control of western blots as indicated. Data are presented as mean ± SEM (*n* = 3), *p < 0.05, **p < 0.01

In order to determine the function of YY1 in human lung epithelial cells, pSG5-YY1 plasmid was transfected into A549 cells. The overexpression of YY1 was confirmed as shown in Fig. 3a. Meanwhile, increased expression of α-sma was observed following YY1 overexpression (Fig. 3b). On the contrary, YY1 shRNA was used to knock down YY1 (Fig. 3c). Consistently, α-sma showed decrease in expression following knockdown of YY1 (Fig. 3d). Upregulation of epithelial marker E-cadherin (Additional file 2 Figure S2A) and downregulation of mesenchymal markers vimentin (Additional file 2 Figure S2B) and N-cadherin (Additional file 2 Figure S2C) were observed in the YY1 shRNA-treated cells.

In addition, EMT and fibrotic markers were analyzed at the protein level in the YY1-overexpressed A549 cells with or without treatment with TGF-β. As seen in Fig. 3e, upregulation of α-sma and snail and downregulation of E-cadherin were observed after YY1 overexpression, which were reinforced by TGF-β stimulation (Fig. 3e). Immunofluorescence staining analysis further demonstrated the increase of α-sma by either YY1 overexpression or TGF-β treatment in A549 cells (Fig. 3f). Quantitative analysis on the fluorescence signal indicated the significant upregulation of α-sma by both YY1 overexpression and TGF-β treatment (Fig. 3 f and Additional file 3 Figure S3).

In order to further validate the pro-fibrotic induction by YY1 in the lung epithelial cells, a human bronchial epithelial cell line BEAS-2B, which is close to type II human AECs, was applied to validate the EMT-promoting function of YY1. As seen in Additional file 4 Figure S4, downregulation of epithelial marker E-cadherin (Additional file 4 Figure S4A) and upregulation of mesenchymal marker slug (Additional file 4 Figure S4B) were accompanied with YY1 overexpression in BEAS-2B cells.

In addition, primary alveolar epithelial cells isolated from the lung tissue of mouse were applied to further validate the YY1 function. The cells were transfected with YY1 plasmid and treated with 5 ng/ml of TGF-β, followed by quantitative real-time PCR analysis of EMT markers including E-cadherin, vimentin and α-sma. As shown in Fig. 3g-i, decrease of E-cadherin expression and increase of α-sma and vimentin expression were observed in the primary epithelial cells after YY1 overexpression and/or TGF-β treatment.

### TGF-β induced translocation of the NF-κB from the cytoplasm to the nucleus

**Fig. 4:**
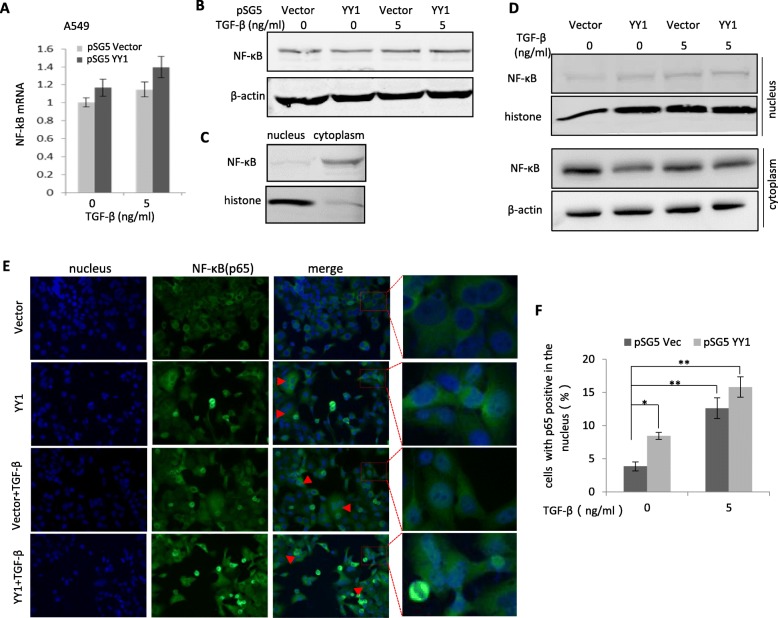
TGF-β induced translocation of the NF-κB protein from cytoplasm to nucleus. **a** and **b** Analyses of NF-κB (p65) at the mRNA level (**a**) or protein level (**b**) in A549 cells treated by 0 or 5 ng/ml of TGF-β with or without overexpression of YY1. Quantitative RT-PCR and western blot were used, respectively. **c** Localization of NF-κB protein in the cytoplasm and the nucleus of A549 cells under normal condition. **d** Analysis of NF-κB in the cytoplasm and the nucleus of A549 cells treated by 0 or 5 ng/ml of TGF-β with or without overexpression of YY1. Western blot was performed. Histone served as a marker for nucleus lysates. β-actin served as a marker for cytoplasm lysates. **e** immunofluorescence staining of A549 cells with antibody against the NF-κB p65 protein to indicate distribution of NF-κB in the cytoplasm and the nucleus. Representative cells with relocalization of NF-κB to the nucleus were indicated with arrows in red. **f** Quantitative analysis of the cells with NF-κB positive in the nucleus in (**e**). Data are presented as mean ± SEM (*n* = 3). *p < 0.05, **p < 0.01

The transcription factor nuclear factor-κB (NF-κB), has been reported to regulate inflammation [27], cystic fibrosis in lung [28], hepatocytes fibrosis in liver [29]. Herein, the expression of NF-κB p65 was examined in A549 cells before and after EMT induction by TGF-β. As shown in Fig. 4a and b, TGF-β treatment did not change the expression level of NF-κB in the lysates from whole cells.

**Fig. 5:**
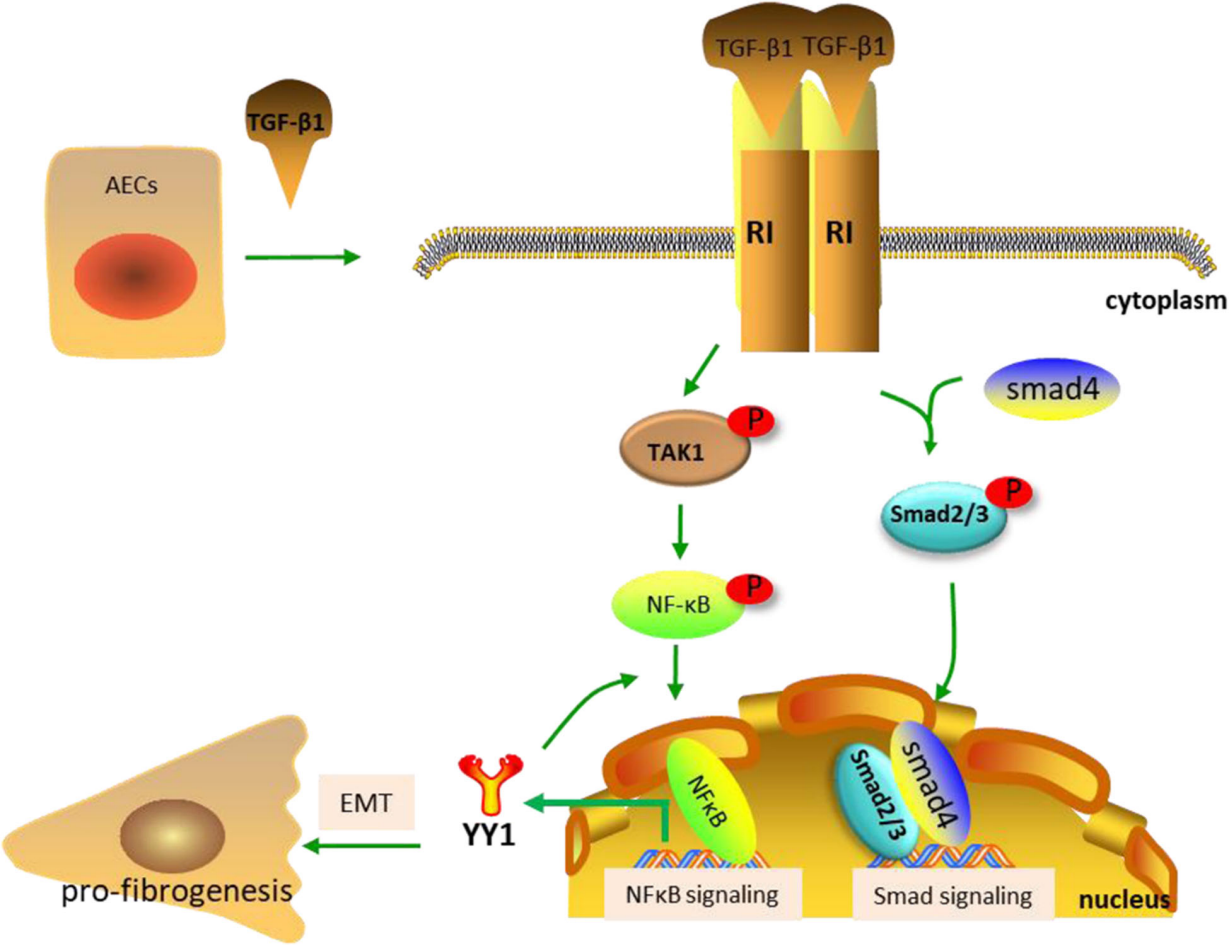
Schematic representation of the hypothetical mechanism by which YY1 is involved in the TGF-β-induced EMT and pro-fibrotic phenotypes in alveolar epithelial cells

The distribution of NF-κB in the cytoplasm and the nucleus were further examined (Fig. 4c). The translocation of NF-κB p65 from the cytoplasm to the nucleus was observed after EMT induction by either YY1 overexpression (Fig. 4d, lane 2 vs lane 1) or TGF-β treatment (Fig. 4d, lane 3 vs lane 1) in A549. The translocation of the NF-κB p65 protein was further validated by immunofluorescence staining (Fig. 4e). Quantitative analysis (Fig. 4f) indicated the significant upregulation of the nuclear NF-κB p65 by both YY1 overexpression and TGF-β treatment. Since nuclear translocation of NF-κB has been confirmed to associate with the pro-fibrotic phenotypes, the current data suggests that cytoplasm to nucleus translocation of NF-κB p65 promotes YY1 expression, which in turn activates NF-κB signaling, mediating TGF-β-induced EMT and fibrosis in the lung epithelial cells (Fig. 5).

## Discussion

Pulmonary fibrosis is an incurable disease associated with lung damage and scarring. In addition to the critical role of the lung fibroblasts and myofibroblasts, emerging evidence indicates the important role lung epithelial cells may play during the pathogenesis of pulmonary fibrosis. Bronchiolar-like epithelial cells and hyperplastic type II AECs lining areas of honeycomb fibrosis have been observed in the pulmonary fibrotic lung biopsies [30]. Furthermore, lung epithelial cells produce pro-fibrotic mediators including connective tissue growth factor (CTGF), platelet derived growth factor (PDGF) and TGF-β [1, 31, 32]. Injury targeted to lung epithelial cells in vivo by Diphtheria toxin increased collagen accumulation in the lung in a mouse model [33]. In the current study, A549, BEAS-2B and primary lung epithelial cells were applied to determine the specific function of YY1 during the pathogenesis of EMT and pro-fibrosis.

It has been reported that a subset of cytokines, called pro-fibrotic factors including TGF-β and tumor necrosis factor-α (TNF-α), are involved in inflammation and fibrosis through different mechanism [34, 35]. Herein, TGF-β was applied to induce pro-fibrotic phenotypes to mimic EMT and pulmonary fibrosis.

As a transcription factor, YY1 is involves in regulating diverse biological processes by activating or repressing gene transcription. YY1 regulates EMT, chemo sensitivity and tumor progression in cancer. YY1 is positively correlated with more malignant phenotype and poorer outcome in several human cancer types [36, 37]. Epithelial expression of YY1 is required in regulating lung branching morphogenesis [38]. In the current study, we demonstrated that YY1 overexpression is required in TGF-β-induced EMT in the human lung epithelial cells. Knockdown of YY1 abrogated the EMT and pro-fibrotic induction by TGF-β. These results indicated a novel function of YY1 in the lung epithelial cells in regulating pulmonary fibrosis. Notably, YY1 showed more enrichment in the primary fibroblast cells from the lung of BALB/C mouse than C57BL/6 mouse. In view of the different Th1/Th2 immune response between the two mouse lines, whether the different levels of YY1 in primary fibroblast cells are related with Th1/Th2 response and pulmonary fibrosis remain to be determined.

The interaction between YY1 and TGF-β is in a gene-specific or signaling pathway-specific manner. A recent publication found the FAM3C-YY1-HSF1 signaling pathway is essential for TGF-β-triggered proliferation and migration of human breast cancer MDA-MB-231 cells [39]. Literature [23, 25] and our current findings demonstrated NF-κB signaling mediates the positive correlation between TGF-β and YY1 in regulating EMT and fibrosis. However, smad signaling has been reported to mediate a negative interaction between YY1 and TGF-β in certain condition. Kurisaki K. et al reported that YY1 inhibited TGF-β-induced cell differentiation through repressing smad transcriptional activities [40]. Yan X. et al found that smad7 interacts with YY1, which can be attenuated by TGF-β signaling [41].

It has been demonstrated that NF-κB/Snail/YY1/RKIP circuitry is involved in the EMT development of cancer cells [23]. Lin X. et al reported that YY1 is overexpressed in fibroblasts in both human IPF and murine models in a NF-κB-dependent manner [25]. Herein, we are the first to find the EMT induction by TGF-β/YY1 signaling in alveolar epithelial cells through nucleic relocalization of NF-κB. Taken together, NF-κB signaling may play important role in regulation of TGF-β/YY1-induced EMT and fibrosis. Although the positive interaction between NF-κB and YY1 has been demonstrated, the regulatory mechanism still needs further exploration.

The critical regulation of TGF-β/NF-κB/YY1 in EMT and tissue fibrosis suggests novel therapeutic targets in treatment of pulmonary fibrotic patients. Several distinct strategies including the administration of anti-TGF-β antibodies or small molecule inhibitors targeting TGF-β are available to inhibit TGF-β signaling. Since the NF-κB signaling has been demonstrated to play an essential role in TGF-β-mediated EMT and/or fibrosis [23, 25, 39], administration of endogenous NF-κB signaling may hold promise in the targeted suppression of tissue fibrogenesis and EMT development. In addition to NF-κB, the current study proved YY1 as a critical mediator for TGF-β-induced EMT and pro-fibrosis in the lung epithelial cells. Targeted inhibition of YY1 alone or combined with TGF-β or NF-κB inhibitors to treat pulmonary fibrosis will provide encouragement for further evaluation.

## Conclusion

Our previous work has determined the regulation of transcription factor YY1 in pathogenesis of fibroid lung. However, the specific function of YY1 in epithelial cells during the pathogenesis of pulmonary fibrosis remains unknown. Here we found that YY1 in lung epithelial cells plays an important role in pathogenesis of lung fibrosis. Upregulation of YY1 and the translocation of NF-κB p65 from the cytoplasm to the nucleus mediate the TGF-β-induced EMT and pro-fibrosis in primary alveolar epithelial cells. These findings will shed light on the better understanding of mechanisms regulating pulmonary fibrotic progression and lung fibrosis.

## Supplementary information

**Additional file 1: Figure S1.** Quantitative RT-PCR analysis of slug (A), claudin-1(B) and ZO-1(C) mRNA level in A549 cells before or after treatment with 5 and 20 ng/ml of TGF-β. TGF-β was applied for 48 h. Data are presented as mean ± SEM (*n* = 3). All comparison was compared with cells without TGF-β treatment. *p < 0.05, **p < 0.01.

**Additional file 2: Figure S2** Quantitative RT-PCR analysis of EMT markers including E-cadherin (A), vimentin (B) and N-cadherin (C) mRNA in A549 cells treated with YY1-shRNA or pLKO vector control under normal culturing condition. Data are presented as mean ± SEM (*n* = 3), *p < 0.05, **p < 0.01.

**Additional file 3: Figure S3.** Quantitative analysis of α-sma immunofluorescence staining in A549 cells treated by 0 or 5 ng/ml of TGF-β with or without overexpression of YY1 as shown in Fig. 3 f.

**Additional file 4: Figure S4.** Quantitative RT-PCR analysis of EMT markers including E-cadherin (A) and slug (B) mRNA in YY1-overexpressed BEAS-2B cells. Data are presented as mean ± SEM (n=3), *p<0.05, **p<0.01.

## Data Availability

All data and materials are available for sharing.

## Abbreviations

AEC: Alveolar epithelial cell
CTGF: Connective tissue growth factor
EMT: Epithelial–mesenchymal transition
FBS: Fetal bovine serum
miRNA: microRNA
PDGF: Platelet derived growth factor
TGF-β: Transforming Growth Factor β
TNF-α: Tumor necrosis factor-α
YY1: Yin Yang1
α-sma: α-smooth muscle actin
