# Supplementary figures and images for: YY1 mediates TGF-β1-induced EMT and pro-fibrogenesis in alveolar epithelial cells
Source: Respir Res. 2019 Nov 8;20:249. doi: 10.1186/s12931-019-1223-7 (PMC6839144; doi:10.1186/s12931-019-1223-7)

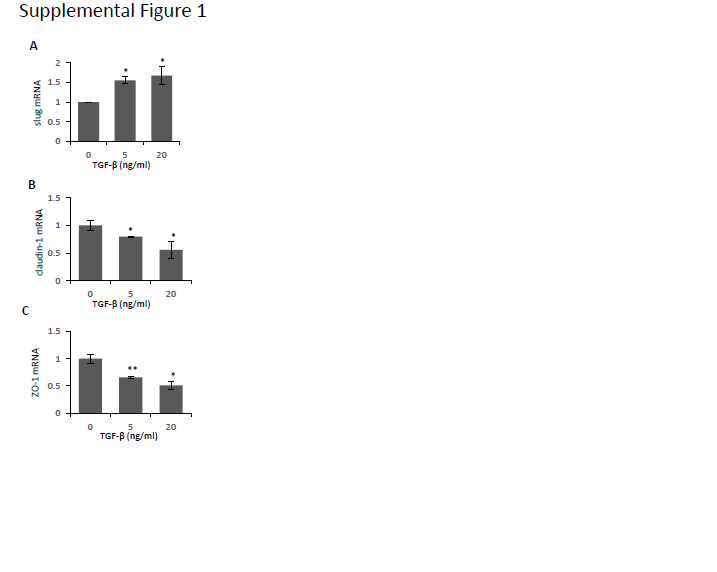

Supplement: Supplementary file 1 — Additional file 1: Figure S1. Quantitative RT-PCR analysis of slug (A), claudin-1(B) and ZO-1(C) mRNA level in A549 cells before or after treatment with 5 and 20 ng/ml of TGF-β. TGF-β was applied for 48 h. Data are presented as mean ± SEM (n = 3). All comparison was compared with cells without TGF-β treatment. *p < 0.05, **p < 0.01. [file 12931_2019_1223_MOESM1_ESM.docx]

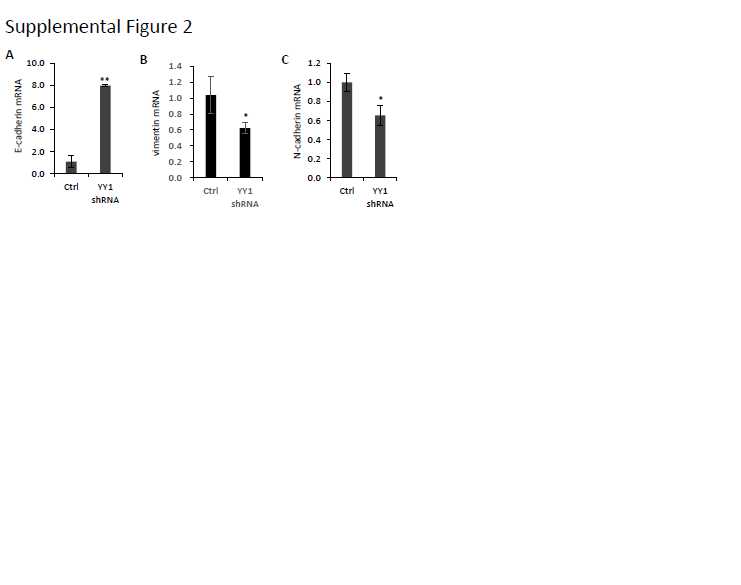

Supplement: Supplementary file 2 — Additional file 2: Figure S2 Quantitative RT-PCR analysis of EMT markers including E-cadherin (A), vimentin (B) and N-cadherin (C) mRNA in A549 cells treated with YY1-shRNA or pLKO vector control under normal culturing condition. Data are presented as mean ± SEM (n = 3), *p < 0.05, **p < 0.01. [file 12931_2019_1223_MOESM2_ESM.docx]

Supplemental Figure 3

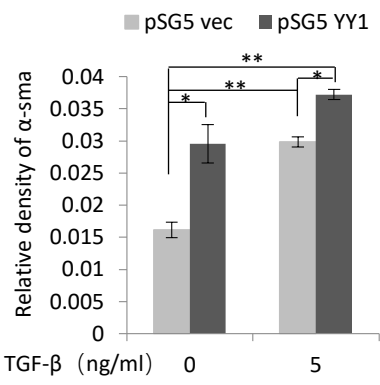

Supplement: Supplementary file 3 — Additional file 3: Figure S3. Quantitative analysis of α-sma immunofluorescence staining in A549 cells treated by 0 or 5 ng/ml of TGF-β with or without overexpression of YY1 as shown in Fig. 3 f. [file 12931_2019_1223_MOESM3_ESM.pdf]

Supplemental Figure 4

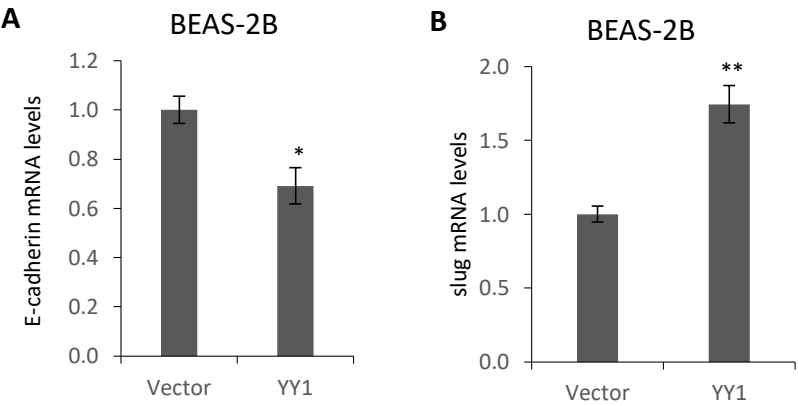

Supplement: Supplementary file 4 — Additional file 4: Figure S4. Quantitative RT-PCR analysis of EMT markers including E-cadherin (A) and slug (B) mRNA in YY1-overexpressed BEAS-2B cells. Data are presented as mean ± SEM (n=3), *p<0.05, **p<0.01. [file 12931_2019_1223_MOESM4_ESM.pdf]
